# MiR-522-3p Targets Transcription Factor 4 to Overcome Cisplatin Resistance of Gastric Cells

**DOI:** 10.1155/2022/6082373

**Published:** 2022-09-27

**Authors:** Guofeng Ma, Wen Xue, Jie Ni, Ran Tao

**Affiliations:** ^1^Department of General Surgery, Nantong Geriatric Rehabilitation Hospital, Nantong 226001, Jiangsu, China; ^2^Department of General Surgery, Affiliated Hospital of Nantong University, Nantong 226001, Jiangsu, China

## Abstract

Gastric cancer (GC) is a malignancy originating from gastric epithelial tissue. Chemoresistance to cisplatin (DDP) often leads to chemotherapy failure in GC. Previously, miR-522 was found to be associated with chemoresistance in GC cells. Thus, we attempted to clarify miR-522-3p's role underlying chemoresistance of GC cells. RT-qPCR measured the miR-522-3p levels in untreated and DDP-treated AGS cells. RT-qPCR and Western blotting detected transcription factor 4 (TCF4) mRNA and protein levels in GC cells. AGS and AGS/DDP cell proliferation were detected by the colony formation assay. Flow cytometry analysis detected AGS and AGS/DDP cell apoptosis. Bioinformatics and dual luciferase reporter assays predicted and verified the relationship between miR-522-3p and TCF4. Rescue experiments further clarified the regulatory patterns of miR-522-3p/TGF4 in GC cells. miR-522-3p presented a downregulation in GC cells and was positively affected by DDP. TCF4 presented elevation in GC cells and was negatively affected by DDP. Mechanistically, miR-522-3p targeted TCF4 to suppress TCF4 gene expression. miR-522-3p overexpression suppressed GC cell proliferation and resistance to DDP and GC cell apoptosis was facilitated. TCF4 overexpression facilitated GC cell proliferation and resistance to DDP while repressing GC cell apoptosis. TCF4 elevation rescued changes in GC cell proliferation, apoptosis, and chemoresistance due to miR-522-3p overexpression. To sum up, miR-522-3p suppresses GC cell malignancy and resistance to DDP via targeting TCF4. Our research may provide a new biomarker for GC diagnosis and a novel direction for GC chemotherapy.

## 1. Introduction

Gastric cancer (GC) is a malignancy that occurs in gastric epithelial tissue [1], and its incidence accounts for 40%–50% of gastrointestinal cancers, ranking first in gastrointestinal tumors [2]. Additionally, its morbidity and mortality rank second among malignant tumors worldwide, and its morbidity and mortality rank first among all cancers in China [2, 3]. At present, surgery is effective for most GC patients [4]. Nevertheless, most of the surgical treatments are carried out in the advanced stages of GC, and the efficacy of surgery alone is very unfavorable [5–7]. Chemotherapy, an important part of comprehensive treatment, has become a vital means of treating GC today. The most effective drugs are 5-fluorouracil, cisplatin (DDP), doxorubicin, etc. [8–10]. Among the chemotherapeutic drugs, DDP is still one of the most commonly used classical drugs for neoadjuvant chemotherapy, postoperative adjuvant chemotherapy, and in vitro drug susceptibility tests for GC patients [11, 12]. However, GC cell resistance to DDP is the main reason for the reduced efficacy of DDP, which has become a major bottleneck for therapy of GC. The existence of resistance to DDP in GC cells reduces the actual efficacy of chemotherapy [13]. Furthermore, the occurrence of drug resistance often leads to chemotherapy failure, thus limiting the clinical application of platinum drugs [14]. Thus, it is urgent to clarify the molecular mechanisms underlying DDP resistance in GC cells.

MicroRNAs (miRNAs) are small noncoding RNAs composed of approximately 19–24 nucleotides in length [15–17], which can modulate target gene expression by degrading target messenger RNA (mRNA) or suppressing mRNA translation [18]. There are gene abnormalities or abnormal expression of miRNAs in a variety of human tumors [19]. miRNAs participate in the regulation of cell proliferation, apoptosis, differentiation, and chemoresistance with the functions of oncogenes and tumor suppressor genes and exert important biological functions in the occurrence and development of tumors [20–22], including GC. For instance, miR-199a-3p facilitates GC cell invasion and migration via downregulating ETNK1 and has an association with poor prognosis [23]. miR-216b suppresses GC proliferation and migration through modulating PARK7 [24]. Previously, microarrays revealed that miR-522-3p was presented as a differential expression in GC [25]. Cancer-associated fibroblasts secrete miR-522, which represses ferroptosis and facilitates chemoresistance in GC cells [26]. Thus, we hypothesized that miR-522-3p may be involved in GC progression.

Herein, we attempted to clarify the role of miR-522-3p and investigated whether miR-522-3p had an association with the chemoresistance of GC cells to DDP. We carried out a series of functional assays in AGS and AGS/DDP cells and also tried to figure out the downstream mechanism of miR-522-3p in GC cells. Our research may provide a new biomarker for GC diagnosis and a novel direction for GC chemotherapy.

## 2. Materials and Methods

### 2.1. Cell Lines, Reagents, and Antibodies

Human gastric mucosal cell line GES-1 (M-C1054) was from Mcellbank (Shanghai, China), GC cell lines (AGS, HGC27 and MKN-45) from ATCC (USA), and DDP-resistant human GC strain AGS/DDP from (JY190) SSRCC (Shanghai, China). Lipofectamine 2000 was from Invitrogen (USA); miR-522-3p mimics and NC mimics from GenePharma (Shanghai, China), transcription factor 4 (TCF4) overexpression vector and empty vector from OriGenl (USA); and TCF4 antibody (ab185736; 1 : 500), GAPDH (ab9485; 1 : 2500), and anti-mouse and anti-rabbit horseradish peroxidaselabeled secondary antibodies from Abcam (Shanghai, China). RPMI1640 culture medium, fetal bovine serum (FBS), and 0.25% trypsin were from Gibco (USA); penicillin and streptomycin from Thermo Fisher Scientific (USA); total RNA extraction reagent Trizol from Thermo Fisher Scientific (USA); and reverse transcriptase kit were from Mingyang Kehua (Beijing, China). SYBR PCR Master Mix kit was from Shanghai Lianmai (Shanghai, China); Annexin V-FITC/PI detection kit was from KeyGEN BioTECH (Jiangsu, China); DDP from Sigma (USA).

### 2.2. Cell Culture

GES-1, AGS, HGC27, MKN-45, and AGS/DDP cells were cultured in RPMI1640 medium containing 10% FBS, 100 U/ml penicillin, and 100 U/ml streptomycin at 37°C, with 5% CO_2_, and cells at the logarithmic growth phase were taken for following assays. DDP was dissolved in normal saline at 4 mg/mL. AGS cells were treated with DDP at different concentrations (0, 5, 10, 15, 20, and 25 *µ*M) for 24 h [13].

### 2.3. RNA Extraction and RT-qPCR

Total RNA was extracted from cells using TRIzol reagent. RNA concentration and purity were determined, followed by stem-loop reverse transcription. The reverse transcriptase kit was used for synthesizing cDNA, followed by PCR amplification. The SYBR PCR Master Mix kit was used for measuring miR-522-3p and TCF4 levels. The primer sequences were as listed: miR-522-3p forward, 5′-GGGCTCTAGAGGGAAGCGC-3′, and miR-522-3p reverse, 5′-CAGTGCGTGTCGTGGAGT-3'; U6 forward, 5′-CTTCGGCAGCACATATACT-3′, and U6 reverse, 5′-AAAATATGGAACGCTTCACG-3'; TCF4 forward, 5′-GGCTATGCAGGAATG TTGGG-3′, and TCF4 reverse, 5′-GTTCATGTGGATGCAGGCTAC-3'; GAPDH forward, 5′-CTGGGCTACACTGAGCACC-3′, and GAPDH reverse, 5′-AAGTGGTCGTTGAGGGCA ATG-3'. The relative expression of miR-522-3p and TCF4 were analyzed using the 2^−△△CT^ method while U6 and GAPDH functioned as the internal controls.

### 2.4. Cell Transfection

AGS and AGS/DDP cells were transfected after 24 h of culture. The cells were cultured to approximately 80% confluence in plates, and then transfected with the indicated miRNA or mRNA plasmids (NC mimics, miR-522-3p mimics, Over-NC, Over-TCF4) using Lipofectamine 2000 according to the manufacturer's instructions. After 48 h of transfection, the cells were harvested for the next experiments.

### 2.5. Colony Formation

AGS and AGS/DDP cells (1 × 10^3^ cells per well) were seeded in a 6-well plate and incubated for 1 week at 37°C. Then, cells were washed twice in PBS, fixed with 4% formaldehyde for 15 min, and stained with crystal violet for 30 min. The colonies (a diameter ≥ 100 *µ*m) were counted in triplicate assays.

### 2.6. Flow Cytometry

The apoptosis was detected by the combined Annexin V-FITC/PI double staining method. AGS and AGS/DDP cells were seeded into cell plates at a density of 5 × 10^4^ cells/well for culture, and then collected within 48 h of transfection. After washing with PBS 3 times, 5 *μ*l of Annexin V-FITC and 10 *μ*l of PI were added, respectively, mixed well and reacted for 10 min at room temperature in the dark. The apoptosis rate was measured on a flow cytometer.

### 2.7. Western Blot

The logarithmic phase AGS cells were taken; the medium in the culture dish was aspirated, and the cells were stored in a sterile centrifuge tube. After centrifugation at 1200 r/min for 10 min, the lysate was added to resuspend the cells. The protein concentration was determined by the BCA method. The 5×SDS gel electrophoresis buffer was added and denatured at 100°C for 10 min. After being completely separated by electrophoresis, the protein was transferred to the PVDF membrane by the semidry method. After blocking with 5% skimmed milk powder at room temperature for 2 h, the specific primary antibodies including GAPDH and TCF4 were added, and incubated overnight at 4°C. The secondary antibodies (1 : 1000) were added, incubated for another 2 h, and washed with TBS. Absorbance analysis was performed after color development to calculate the relative expression of each protein. The chemiluminescence reagent was added to band development, with GADPH as an internal reference. Quantity One®4.62 software (BioRad, USA) was used to analyze band intensity.

### 2.8. Bioinformatics

A downstream molecule of miR-522-3p was predicted by starBase 2.0 (https://starbase.sysu.edu.cn/agoClipRNA.php?source=mRNA) with conditions (CLIP Data: strict stringency; Degradome Data: high stringency; Pan-Cancer: 6 cancer types). The binding sequence of miR-522-3p on TCF4 3′untranslated region (UTR) was also predicted by starBase 2.0 website.

### 2.9. Luciferase Reporter Assay

The 3′UTR sequence and the mutant sequence containing the complementary site of the target gene and miRNA were amplified. The 5′ end of the upstream and downstream primers each contained different restriction sites. The target band was detected by electrophoresis; the size of the band was observed; the PCR product was purified using the kit for later use; and the ligation reaction mixture was prepared. After inoculation/transfection, 1 × Passive Lysis Buffer, 20 *μ*l/well, was added to the 96-well plate. The lysed AGS cells were pipetted repeatedly to aspirate 15 *μ*l, added to the luciferase assay substrate and mixed well. The data were detected and recorded at 500 nm by a microplate, and the ratio of the two measured data represented the relative fluorescence intensity of samples in each well.

### 2.10. Statistical Analysis

The SPSS 20.0 software was used to process the data. The data were expressed as the mean± standard deviation. The mean of samples between two groups was compared using a *t*-test, and that of multiple groups using one-way analysis of variance followed by Tukey's post hoc test. The difference was statistically significant at *p* < 0.05.

## 3. Results

### 3.1. miR-522-3p Presents Downregulation and Is Positively Regulated by DDP in GC Cells

**Figure 1:**
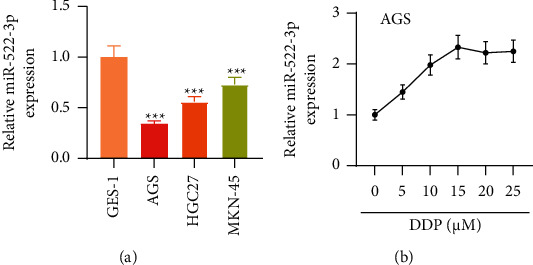
miR-522-3p presented downregulation in GC cells. (a) RT-qPCR measured miR-522-3p level in control cells and GC cell lines. (b) RT-qPCR detected miR-522-3p level in AGS cells under DDP treatment at different doses. ^*∗∗∗*^*p* < 0.001, AGS, HGC27, MKN-45 vs. GES-1 group.

Previously, miR-522-3p showed aberrant expression in GC cells [25]. Nevertheless, its biological role in GC cells remains elusive. Thus, we first determined miR-522-3p expression status in GC cell lines. RT-qPCR demonstrated that miR-522-3p presented depletion in GC cell lines (AGS, HGC27 and MKN-45) relative to normal control cell line GES-1 (*p* < 0.05). Additionally, miR-522-3p showed the most downregulation in AGS cells (Figure 1(a)). Thus, AGC cells were chosen for the following assays. Then, we clarified whether miR-522-3p expression had an association with DDP in GC cells. Thus, we stimulated AGS cells with DDP at different concentrations (0, 5, 10, 15, 20, and 25 *μ*M). RT-qPCR illustrated that miR-522-3p level presented a dose-dependent elevation along with DDP concentration increasing from 0 *μ*M to 15 *μ*M (*p* < 0.05) and then showed no significant changes from 15 *μ*M to 25 *μ*M in AGS cells (*p* > 0.05). miR-522-3p presented a peak level under 15 *μ*M of DDP stimulation in AGS cells (Figure 1(b)). Collectively, miR-522-3p presents depletion and is positively affected by DDP in GC cells.

### 3.2. miR-522-3p Inhibited GC Cell Resistance to DDP in Vitro

**Figure 2:**
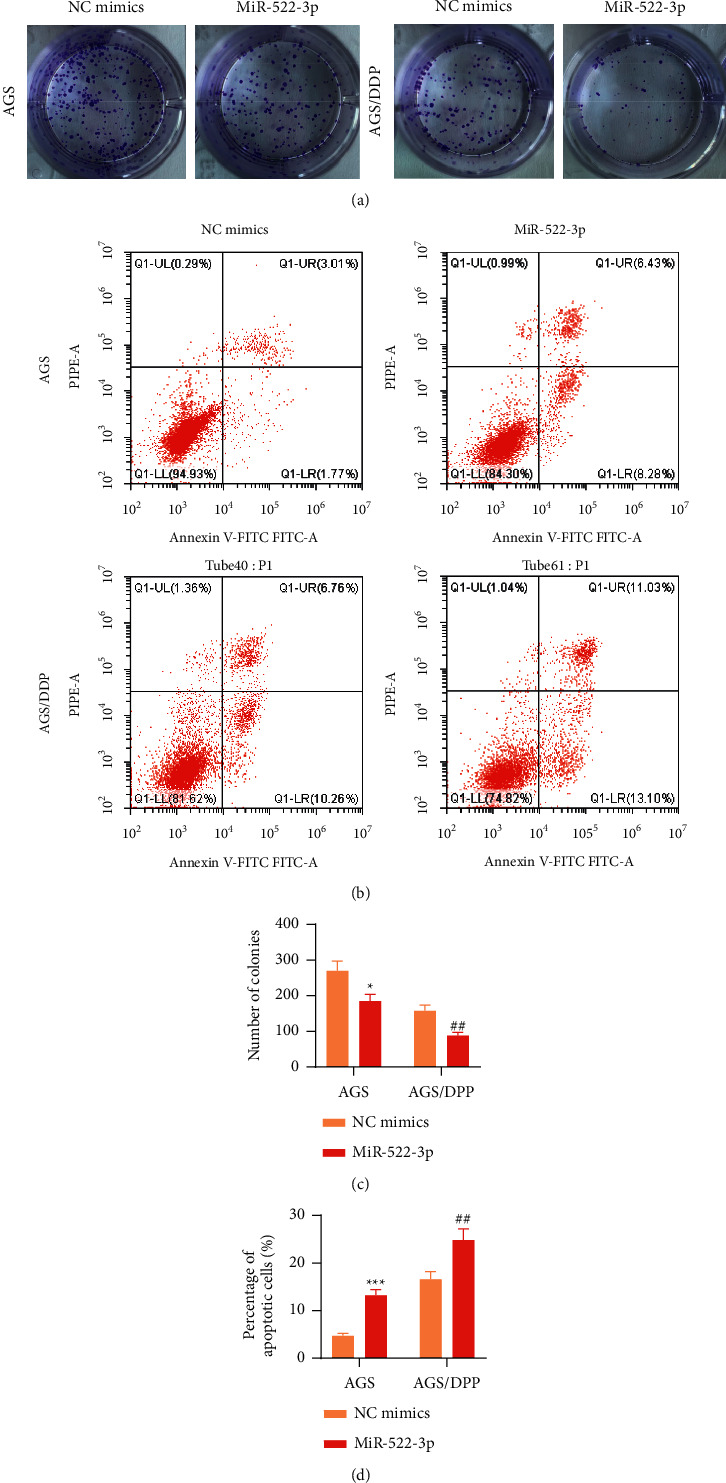
miR-522-3p facilitated GC cell resistance to DDP. (a) Colony formation assessed AGS and AGS/DDP cell proliferation under indicated transfection. (b) Flow cytometry evaluated AGS and AGS/DDP cell apoptosis under indicated transfection. (c) Quantification of number of colonies in AGS and AGS/DDP cells under indicated transfection. (d) Quantification of proportion of apoptotic AGS and AGS/DDP cells under indicated transfection. ^*∗*^*p* < 0.05, ^*∗∗*^*p* < 0.01, ^*∗∗∗*^*p* < 0.001, miR-522-3p vs. NC mimics group; ^##^*p* < 0.01, miR-522-3p vs. NC mimics group.

To determine whether miR-522-3p conferred chemoresistance in GC cells, AGS cells and human DDP-resistant strain AGS/DDP cells, both received transfection with NC mimics plasmid or miR-522-3p mimics plasmid for 48 h. Then, we conducted a series of gain-of-function assays in vitro. As a result, the number of colonies showed a marked decrease in AGS cells under miR-522-3p overexpression (*p* < 0.05), indicating that miR-522-3p suppressed GC cell proliferation. AGS/DDP cell proliferation showed a similar trend under miR-522-3p overexpression (*p* < 0.05, Figures 2(a) and 2(c)), indicating that DDP-resistant GC cells with miR-522-3p overexpression were more sensitive to DDP. Furthermore, miR-522-3p upregulation accelerated AGS cell apoptosis (*p* < 0.05), and similar results were observed in AGS/DDP cells under miR-522-3p upregulation (*P* < 0.05, Figures 2(b) and 2(d)). Collectively, miR-522-3p suppresses GC cell malignancy and GC cell resistance to DDP in vitro.

### 3.3. miR-522-3p Targets TCF4 in GC Cells

**Figure 3:**
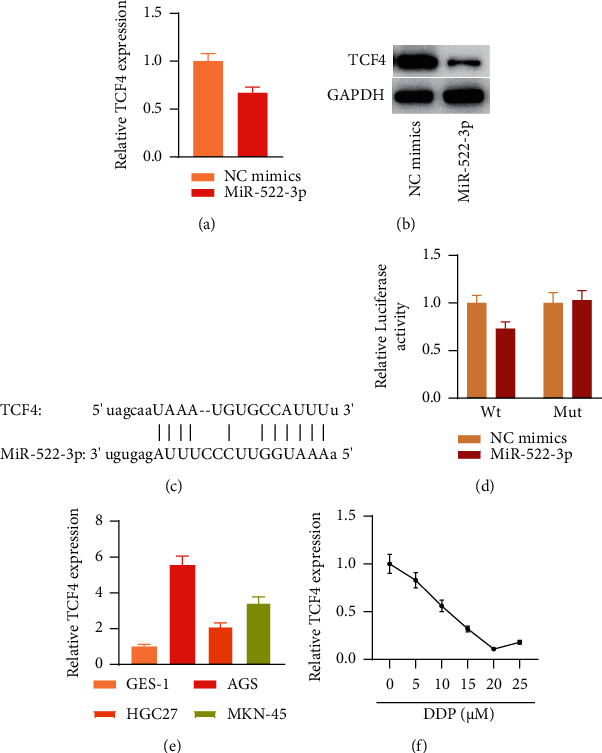
miR-522-3p targeted TCF4 in GC cells. (a) RT-qPCR measured TCF4 mRNA level in AGS cells under NC mimics or miR-522-3p mimics transfection. ^*∗*^*p* < 0.05, miR-522-3p vs. NC mimics group. (b) Western blotting detected miR-522-3p protein level in AGS cells under NC mimics or miR-522-3p mimics transfection. (c) starBase predicted binding fragment of miR-522-3p on TCF4 3′UTR. (d) Luciferase reporter assay assessed relationship of miR-522-3p and TCF4 in GC cells. ^*∗*^*p* < 0.05, miR-522-3p vs. NC mimics group. (e) RT-qPCR measured TCF4 level in control cells and GC cell lines. ^*∗∗∗*^*p* < 0.001, AGS, HGC27, MKN-45 vs. GES-1 group. (f) RT-qPCR detected TCF4 level in AGS cells under DDP treatment at different doses.

MiRNAs exert regulation of target gene expression via degradation of target mRNA or suppressing mRNA translation [18]. We attempted to clarify whether miR-522-3p exerts its role in GC cells in such a manner. Through bioinformatics using starBase, TCF4 was predicted as a putative target of miR-522-3p. RT-qPCR results revealed that miR-522-3p overexpression led to TCF4 depletion in AGS cells (*p* < 0.05, Figure 3(a)). Western blotting results showed a similar trend at the protein level (*p* < 0.05, Figure 3(b)). The binding sequence of miR-522-3p on TCF4 3′UTR was obtained from starBase (*p* < 0.05, Figure 3(c)). After mutation of the binding sequence, we conducted a luciferase reporter assay to determine the relationship between miR-522-3p and TCF4. The results depicted that miR-522-3p elevation suppressed luciferase activity of TCF4 3′UTR-Wt whereas had no influence on luciferase activity of TCF4 3′UTR-Mut in GC cells (*p* < 0.05, Figure 3(d)). Moreover, RT-qPCR demonstrated that TCF4 presented upregulation in GC cell lines relative to normal control cell line GES-1 and AGS expressed the most TCF4 among GC cell lines (*p* < 0.05, Figure 3(e)), which suggested that TCF4 may exert an oncogene in GC cells. Additionally, TCF4 level showed a dose-dependent decline along with DDP concentration increasing from 0 *μ*M to 20 *μ*M (<0.05) and then presented no significant changes from 20 *μ*M to 25 *μ*M in AGS cells (*p* > 0.05). TCF4 expressed at the lowest level under 20 *μ*M of DDP stimulation in AGS cells (Figure 3(f)). Collectively, miR-522-3p or DDP negatively modulates TCF4 level and TCF4 level presents elevation in GC cells.

### 3.4. TCF4 Enhances GC Cell Resistance to DDP in Vitro

**Figure 4:**
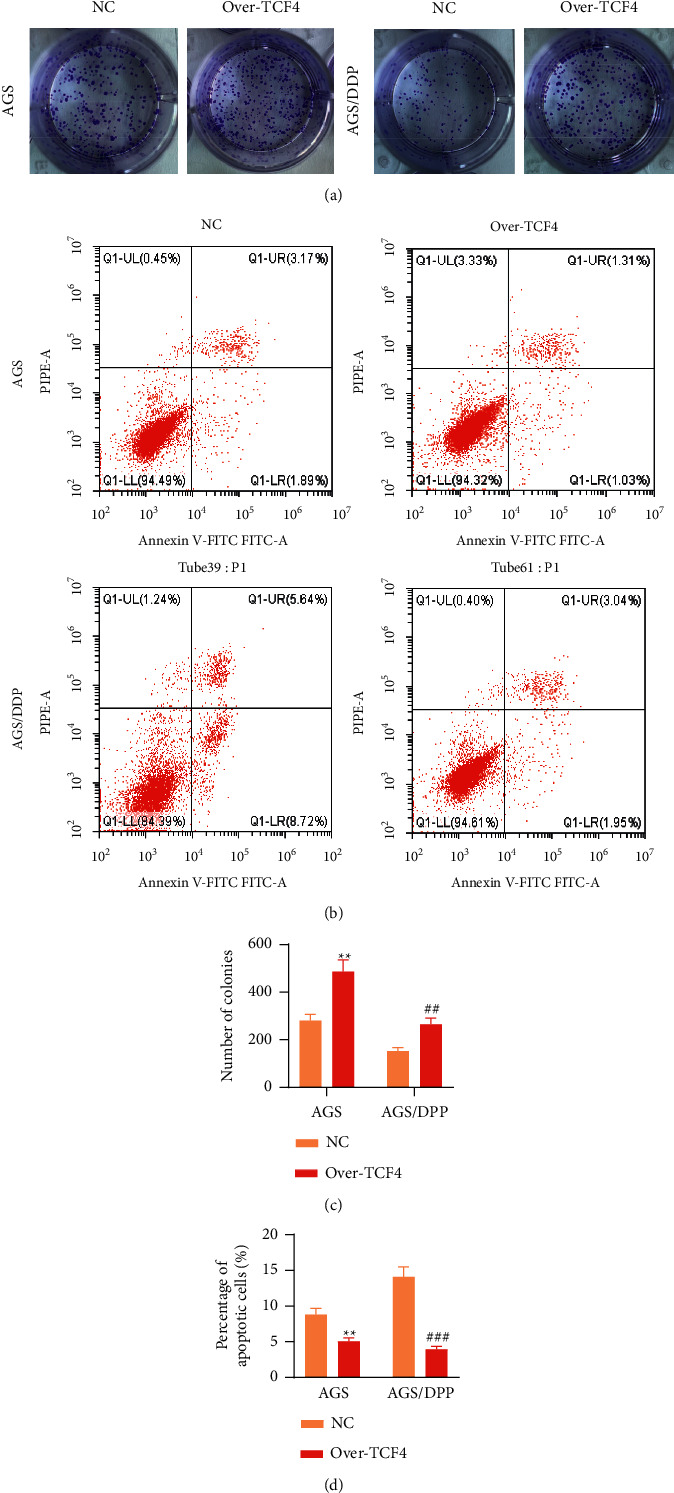
TCF4 suppressed GC cell resistance to DDP. (a) Colony formation assessed AGS and AGS/DDP cell proliferation under indicated transfection. (b) Flow cytometry evaluated AGS and AGS/DDP cell apoptosis under indicated transfection. (c) Quantification of number of colonies in AGS and AGS/DDP cells under indicated transfection. (d) Quantification of proportion of apoptotic AGS and AGS/DDP cells under indicated transfection. ^*∗∗*^*p* < 0.01, ^*∗∗∗*^*p* < 0.001, Over-TCF4 vs. NC group; ^##^*p* < 0.01, ^###^*p* < 0.001, Over-TCF4 vs. NC group.

To clarify whether TCF4 exerted chemoresistance in GC cells, AGS cells, and AGS/DDP cells, both received transfection with an empty vector or TCF4 overexpression vector for 48 h. Then, we conducted a series of gain-of-function assays in vitro. As a result, an amount of colonies showed a marked elevation in AGS cells under TCF4 overexpression (*p* < 0.05), indicating that TCF4 facilitated GC cell proliferation. AGS/DDP cell proliferation showed a similar trend under TCF4 overexpression (*p* < 0.05, Figures 4(a), and 4(c)), indicating that DDP-resistant GC cells with TCF4 upregulation were more resistant to DDP. Furthermore, TCF4 upregulation suppressed AGS cell apoptosis (*p* < 0.05), and similar results were observed in AGS/DDP cells under TCF4 upregulation (*p* < 0.05, Figures 4(b) and 4(d)). Collectively, TCF4 facilitates GC cell malignancy and GC cell resistance to DDP in vitro.

### 3.5. miR-522-3p Overcomes GC Cell Resistance to DDP via Targeting TCF4

**Figure 5:**
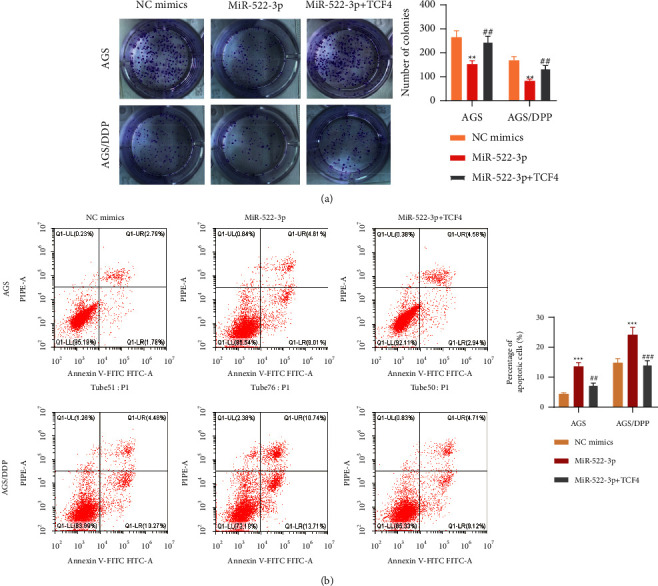
MiR-522-3p facilitated GC cell resistance to DDP via targeting TCF4. (a) Colony formation assessed AGS and AGS/DDP cell proliferation after indicated treatment. (b) Flow cytometry evaluated AGS and AGS/DDP cell apoptosis after indicated treatment. ^*∗∗*^*p* < 0.01, ^*∗∗∗*^*p* < 0.001, miR-522-3p vs. NC mimics group; ^##^*p* < 0.01, ^###^*p* < 0.001, miR-522-3+TCF4 vs. miR-522-3p group.

Finally, to further validate our hypothesis of a regulatory pattern between miR-522-3p and TCF4 in GC cells, we carried out rescue experiments by cotransfection of NC/miR-522-3p mimics and TCF4 overexpression vector in AGS or AGS/DDP cells. After 48 h, TCF4 upregulation neutralized the influence of miR-522-3p on proliferation and apoptosis of AGS cells and AGS/DDP cells (*p* < 0.05, Figure 5(a) and 5(b)). Collectively, miR-522-3p hinders GC cell malignancy and GC cell resistance to DDP via targeting TCF4.

## 4. Discussion

In recent years, the roles of miRNAs in the occurrence and development of malignancies have received extensive attention. miRNAs may act as oncogenes or tumor suppressor genes in tumors [27–29]. Herein, miR-522-3p showed great downregulation in GC cell lines, suggesting that miR-522-3p may exert a tumor suppressor in GC cell behaviors.

Tumors are the leading causes of death globally, killing nearly 10 million people [30, 31]. In addition to controlling the main key parameters of cancer therapy management, such as diagnosis, resistance to both classic and new chemotherapeutic agents remains a significant problem [32]. In many cases, intrinsic or acquired chemoresistance leads to cancer recurrence, ultimately resulting in failure of successful treatment and death in cancer patients [32]. Various determinants, including tumor heterogeneity and the tumor microenvironment, can induce chemoresistance through multiple mechanisms [33]. Platinum drugs, especially cisdiaminedichloroplatinum (II) (the best known DDP), are applied to treat a variety of solid malignancies, including testicular, ovarian, head and neck, colorectal, and bladder cancers, and lung cancer, etc. [34]. DDP exerts an anti-tumor role through multiple mechanisms. Despite consistent initial response rates, DDP therapy often leads to chemoresistance development, causing treatment failure [34]. The miRNAs can exert regulation of GC cell chemoresistance to DDP. For instance, exosome-derivedmiR-21 confers DDP resistance in GC cells [14]. miR-873-5p exerts function on modulation cellular processes and regulating chemoresistance in GC [35]. Targeting oncogenic miR-181a-2-3p suppresses GC cell malignant behaviors and represses resistance to DDP [36]. Moreover, cancer-associated fibroblasts secrete miR-522, which represses ferroptosis and facilitates chemoresistance in GC cells [26]. Herein, miR-522-3p suppressed GC cell proliferation and elevated GC cell apoptosis. Additionally, miR-522-3p overexpression reversed GC cell resistance to DDP, which is consistent with previous reports.

Gene expression alternation is a major molecular mechanism responsible for the pathological process of human diseases such as tumors [37]. MiRNAs actually get involved at the post-transcriptional level and bind to the target mRNA 3′UTR to inhibit expression [37]. Herein, through bioinformatics, TCF4 was predicted as a putative downstream molecule of miR-522-3p. Mechanistically, miR-522-3p targeted TCF4 3′UTR and repressed its translation, thereby leading to TCF4 downregulation at both mRNA and protein levels. Previously, TCF4 presented elevation in the GC cells, higher levels of TCF4 indicated poorer prognosis of GC, and miR-133a-5p functioned as a GC tumor suppressor through targeting TCF4 [38]. Herein, TCF4 presents upregulation in the GC cell line AGS cells. Moreover, TCF4 served as an oncogene via promoting GC cell malignancy. It has been revealed that H19 suppresses chemosensitivity of GC cells to adriamycin via binding to miR-152 and targeting TCF4, leading to suppression of EMT [39]. The IPA network analysis has revealed coordinate elevations of DKK1 transcriptional regulators, including TCF4 in the DDP-surviving clones [40]. Herein, TCF4 overexpression promoted GC cell resistance to DDP. Furthermore, through rescue assays, we further validated that TCF4 elevation rescued the changes in proliferation, apoptosis, and chemoresistance of DDP under miR-522-3p overexpression in GC cells.

In conclusion, miR-522-3p suppresses GC cell malignancy and GC cell resistance to DDP via targeting TCF4, providing a new biomarker for GC diagnosis and a novel direction for GC chemotherapy.

## Data Availability

The data analyzed in the present study can be obtained from the corresponding author upon reasonable request.
